# Georadar Waveform Characterization of Tunnel Lining Rear Defects and Joint Detection Method in Time and Frequency Domains

**DOI:** 10.3390/s25227086

**Published:** 2025-11-20

**Authors:** Jian Liu, Wei Yan, Gaohang Lv, Lei Kou, Bo Li, Xiao Zhang, Guanhong Lu, Quanyi Xie

**Affiliations:** 1 School of Qilu Transportation, Shandong University, Jinan 250014, China; lj75@sdu.edu.cn (J.L.); 202435516@mail.sdu.edu.cn (W.Y.); lvgh1125@163.com (G.L.); lei.kou@sdu.edu.cn (L.K.); 202215404@mail.sdu.edu.cn (G.L.); 2 Shandong Research Institute of Industrial Technology, Jinan 250014, China; 3 School of Resources and Environmental Engineering, Guizhou University, Guiyang 550025, China; 4 Laoshan Laboratory, Qingdao 266200, China; sduzhangxiao@sdu.edu.cn; 5 Weifang Hydrodynamics Science and Technology Industry Institute, Weifang 261200, China

**Keywords:** ground-penetrating radar, tunnel lining structure, defect detection, waveform characteristics, time–frequency-domain analysis

## Abstract

Aiming at the signal interference and feature recognition difficulties existing in the detection of concealed defects such as cracks and voids behind the tunnel lining, this study carried out a 1:1 reinforced concrete–steel arch frame composite lining model test; simulated the surrounding rock defects scenarios of three types of filling media, namely crushed stone, air, and water; and analyzed the time-domain, frequency-domain, and time–frequency-domain characteristics of the geological radar signal data. The research finds that the water-filled area generates a strong reflection due to the high dielectric constant, with the spectral peak reaching 712 MHz and the high-frequency component significantly enhanced. The peak frequency of the air-filled zone spectrum is 531 MHz, and the high-frequency bandwidth is broadened. The spectral peak of the crushed stone filling area is 507 MHz, with fast high-frequency attenuation and energy dispersion. The time-domain waveforms show that the amplitude in the water-filled area is the highest and the tailing is obvious, the waveform in the air-filled area is sharp, and the amplitude in the crushed stone-filled area is gentle. The peak frequency of the spectrum, the amplitude attenuation law, and the waveform shape can be used as the key indicators for discriminating the category of filling materials. The analysis method of feature fusion in the time–frequency domain has important engineering application value for improving the detection accuracy of geological radar in complex lining structures.

## 1. Introduction

The “14th Five-Year Plan for the Development of Highways” released by the Ministry of Transport of China has clarified the overall thinking for the development of highway transportation during the “14th Five-Year Plan” period. It is not only necessary to increase construction efforts, but also to attach importance to the development of multiple aspects such as management, maintenance, and operation. China is the country with the largest number of highway tunnels in the world, the most complex geological conditions and structural forms, and the fastest development speed. By the end of 2023, there were a total of 27,297 highway tunnels in China, with a total length of 30.2318 million meters, and 205 extra-long tunnels, with a total length of 9.2407 million meters. There are 18,573 railway tunnels put into operation, with a total length of 23,508 km. With the rapid development of tunnel construction, the primary issue that comes along is the maintenance and repair of tunnels [1], and the defect detection of tunnel linings is the cornerstone of this work.

The defects of tunnel linings mainly focus on the apparent cracks of the linings and the fissures and voids behind them. The traditional detection and identification of cracks are mainly carried out through manual inspection and manual marking. At present, the common method for detecting fissures and cavities behind tunnel linings at home and abroad is ground-penetrating radar (GPR) [2,3], which can detect the distribution, structural morphology, and variation characteristics of underground materials efficiently and accurately. Wu Xianlong et al. [4] established a forward model based on the FDTD method to simulate the detection process and analyzed the responses of different-sized air and water voids in ordinary concrete linings and reinforced concrete linings. Zhang Liang et al. [5] conducted forward simulation and on-site test analysis using the GPRMax software, and detected various fillers in cavities of different shapes through ground radar. Jianbo [6] conducted on-site simulation tests of geological radar to study the characteristics of geological radar waves under three conditions: no water accumulation around the tunnel lining, local water accumulation, and complete water accumulation. Liu Hai et al. [7] studied the influence of steel mesh in reinforced concrete structures on the electromagnetic wave scattering and penetration characteristics of GPR through numerical and laboratory experiments. Qin Chengke [8] studied the application of the method combining ground-penetrating radar detection and numerical analysis in the quality inspection of tunnel lining structures. Wang Jinbiao [9] discussed and summarized the image features of targets such as reinforcing bars and arch frames in tunnel linings. Wen Shiru et al. [10] proposed a spectral energy interpretation method based on Counterlet isometric transform and K-means++ clustering to address the problem of strong subjectivity in manual interpretation of geological radar images. To solve the problems such as the difficulty in accurately simulating complex defects in tunnel linings, many researchers [11,12,13] have utilized deep learning models such as Segnet and GPRInvNet, and the TPE-CatBoost machine learning method, to achieve defect segmentation, dielectric constant reconstruction, and classification.

Although most researchers are striving to extract the features of georadar images in order to be able to quickly and accurately determine the defects of tunnel lining structures, the waveforms displayed by the electromagnetic waves of georadar when passing through different media vary greatly. Moreover, the steel mesh and steel arch frame structures inside the tunnel lining have a relatively serious signal shielding effect on electromagnetic waves [14]. This brings difficulties to the feature extraction and discrimination of the defects behind the tunnel lining. Therefore, noise reduction processing and rapid imaging of ground-radar data have become the research directions of most scholars. Lv Gaohang et al. [15] analyzed the differences in characteristic parameters such as amplitude and signal entropy between defect and non-defect GPR data through model testing. Based on the differences, they proposed an automatic GPR identification method and compiled the program in MATLAB R2022b. Zheng Yifeng et al. [16], from the measurement perspective, reduced the interpolation space without using the measuring wheel to improve the axial accuracy of the test data. Lin Chunjin et al. [17] classified the defects of tunnel lining, such as cracking, voids, and leakage, and conducted numerical experiments using ground-penetrating radar combined with the finite-difference time-domain (FDTD) method. Yu Cong et al. [18] carried out the migration processing of GPR data by using the F-K offset theory and inverse time offset, providing convenience for the analysis of radar data. Zhu Ziqiang [19] used S-transform time–frequency processing to handle simulation data and actual data, providing an effective solution to the disturbance of tunnel lining data. Li Dongli et al. [20] proposed an improved GPR data processing method combining backprojection (BP) imaging with robust principal component analysis (RPCA), through which the shape and position of defect responses were restored by BP, and the target and clutter were isolated by RPCA. Aiming at the problem that the shielding of the reinforcing bar layer makes it difficult for GPR to identify the gap defects beneath it, most scholars [21,22,23,24,25,26] have proposed the unsupervised generative network model based on VAE and GAN, SA-DenseCL self-supervised learning, RDCNN-D-2/YOLOv8 deep learning model, and M-YOLACT multi-task network. Combining technologies such as feature extraction and signal suppression, the accuracy and automation level of defect detection are improved. Meanwhile, many studies have explored advanced processing and interpretation methods for ground-penetrating radar (GPR) signals in tunnel lining inspection. Most of these studies [27,28,29,30,31] focus on algorithm optimization and learning-based defect recognition techniques, such as deep learning detection and full-waveform inversion models.

In conclusion, although the above-mentioned research has improved imaging accuracy and the level of automation, the verification mainly relies on simulation or small-scale laboratory tests. In contrast, there is a lack of high-fidelity experimental research to quantitatively analyze the radar response under actual tunnel lining conditions. To make up for this gap, this paper conducts 1:1 scale physical model experiments, integrating time-domain and frequency-domain analyses, to provide reliable waveform indicators for actual tunnel detection.

## 2. Geological Radar Lining Prototype Tests

### 2.1. Test Device

In actual engineering, due to the good condition of the surrounding rock, people often tend to overlook the cracks and voids behind the secondary lining. The structural damage caused to the tunnel lining may, thereby, directly lead to construction accidents. Therefore, in this experiment, by simulating the actual situation of the tunnel lining, the caves with different fillers behind the lining were detected, and the corresponding defect radar waveforms were classified and summarized.

**Figure 1:**
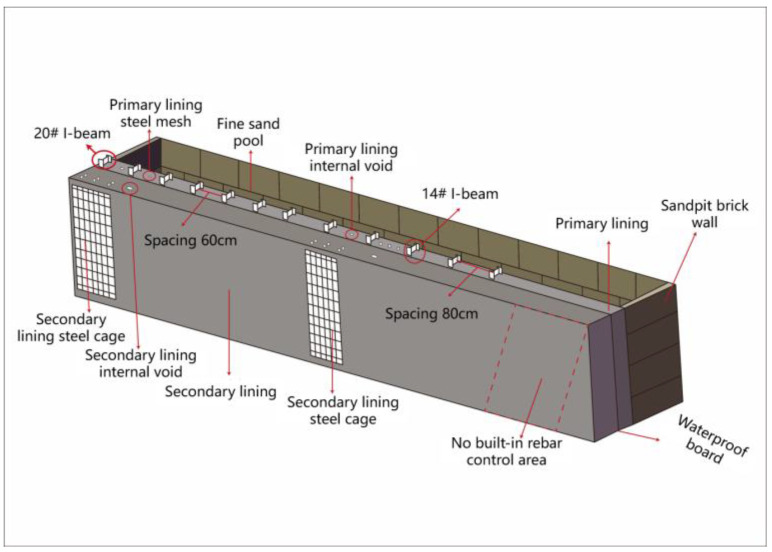
Lining prototype test drawing.

The design of the test model is shown in Figure 1 below. The model used reinforced concrete as the main structural material and was fabricated in combination with steel arch frames to realistically reproduce the defect distribution of the lining structure in actual engineering at a one-to-one scale. In the initial support part of the model, steel mesh sheets and 14# and 20# I-beams were added, and cracks were set inside. In the secondary lining, two steel cages and internal voids were set up, and a comparison area without built-in steel bars was reserved. After the initial support, a sandpit was arranged to simulate the surrounding rock. In the sandpit, PVC pipes of different diameters were used to simulate cavity defects, and water and crushed stones were filled inside them.

### 2.2. Test Defect Simulation Method

The test model adopted a 1:1 scale reinforced concrete–steel arch frame composite structure, with multiple types of defects implanted layer by layer to simulate the defect characteristics of actual tunnel linings (Figure 1). The model construction is divided into three subsystems: primary support defects, secondary lining defects, and surrounding rock defects. The specific simulation methods are as follows:

(1) Simulation of initial support defects

I-beam frames were arranged at the designed intervals within the shotcrete layer (Figure 1), and an alternating arrangement was adopted to balance the stiffness distribution of the structure. The steel mesh was formed into a uniformly stressed grid through cross-welding. The strength of the nodes and the lap length strictly followed the engineering specifications to ensure the authenticity of the initial mechanical properties of the support system. The initial structural damage was constructed by presetting artificial cracks, and the surface of the prefabricated cracks was roughened. The microscopic morphological characteristics formed by oxidation erosion were reproduced by the sandblasting process.

(2) Simulation of secondary lining defects

The simulation of secondary lining defects focused on voids and reinforcement shielding effects, and realized the controllable implantation of multiple types of defects through systematic process design. Firstly, within the lining model, the double-piece steel cage reinforcement area and the unreinforced comparison area were divided. The steel cage was formed by binding the circumferential main bars and stirrups. The spacing of the main bars and the thickness of the protective layer were set in equal proportions according to the actual engineering standards. The same concrete pouring conditions were retained in the unreinforced areas to form a comparison benchmark for mechanical properties. Secondly, during the concrete pouring stage, a rectangular cavity was formed by presetting detachable formwork. The size and position of the cavity were calibrated based on the statistics of typical engineering defects. After the formwork was removed, a smooth interface was retained to simulate the de-bonding phenomenon between the lining and the surrounding rock, and a transition zone was set at the edge of the cavity to weaken the boundary effect caused by artificial defects. Finally, for the unreinforced area, the stratified vibration and aggregate regulation process was adopted to artificially introduce the porosity gradient distribution within the local range. By adjusting the vibration time and aggregate gradation, a progressive transition from the dense zone to the loose zone was formed to reproduce the characteristics of material performance deterioration caused by uneven construction quality.

(3) Simulation of surrounding rock defects

The surrounding rock matrix was prepared by using graded sandy soil according to the engineering analogy method. The uniform distribution of density and moisture content was achieved through stratified compaction and humidity control to simulate the actual stratum’s mechanical characteristics. Sandpit space on the outside of the lining model was reserved to ensure that the area where the defect is implanted maintains geometric compatibility with the lining structure. The PVC pipe cavity model was laid out based on the three-dimensional coordinate system, and the center position and burial depth were calibrated. The diameters of the cavities were set hierarchically in small, medium, and large gradients, and the burial depth covered the contact surface to the deep area to construct the void effect at different spatial positions. Taking the cavity size, burial depth, and filling medium as the basic variables, combined with different supporting structure parameters (stiffness of I-beams, density of steel mesh), a full-factor experimental matrix was formed. The working condition combination was optimized through the orthogonal test method to cover the typical engineering scenarios of single defect to multiple defect coupling.

The experimental design was based on the actual engineering defect statistics data. The orthogonal experiment method was adopted to generate the full-factor working conditions (see Table 1). Combined with the multi-frequency scanning of ground-penetrating radar and high-precision spatial positioning, multi-dimensional data covering geometric parameters, material properties, and signal characteristics were formed. This model achieves a progressive simulation from a single defect to a composite defect and from local damage to system response through hierarchical defect implantation and multi-physics field coupling, providing a high-confidence experimental benchmark for the verification of non-destructive testing techniques for concealed tunnel defects.

### 2.3. Test Acquisition Equipment

This experiment took the LTD ground-penetrating radar system as the core detection equipment. Its radar host was equipped with a 400 MHz/900 MHz dual-frequency combined antenna. The low-frequency 400 MHz antenna enhanced the penetration ability of deep signals, and the high-frequency 900 MHz antenna optimized the resolution of shallow structures. The sampling frequency was set to 100 GHz and supported flexible adjustment of the 5–100 ns time window. Among them, the 400 MHz antenna defaulted to using a 60 ns time window to cover the detection depth range of 0–3 m. The gain mode adopted a combination of automatic optimization and manual segmented adjustment, with a dynamic range of no less than 160 dB, and integrated a time-varying gain compensation function to adapt to the attenuation characteristics of the medium. The trigger system achieved millimeter-level displacement synchronization accuracy based on the photoelectric coding wheel, ensuring the spatio-temporal consistency of the scanning trajectory and data acquisition.

The auxiliary positioning system was composed of a rangefinder and a total station working together. The rangefinder was used for precise calibration of the defect burial position and the burial depth calibration of PVC pipes, while the total station recorded the three-dimensional spatial coordinates of the survey line. The planar positioning error was controlled within ±2 mm, and the elevation error was ±3 mm, achieving seamless association between radar data and geographic information.

The defect simulation tool included three types of dedicated devices: The PVC cylinders were available in three specifications with outer diameters of 7.5 cm, 16 cm, and 25 cm, and the bottom was sealed and anti-seepage-treated to simulate a closed cavity. The water injection device was equipped with a variable-frequency electric water pump (flow rate 5 L/min, pressure 0.2 MPa) and a water level sensor. The crushed stone filling adopted limestone with a particle size of 5–10 mm, and was compacted layer by layer with a 50 Hz vibrator to reproduce the mechanical properties of the loose medium.

### 2.4. Test Collection Process

Before the experiment began, the LTD-type ground radar system was assembled, and the antenna frequency, time window range, and gain parameters were debugged. By calibrating the signal reflection intensity and noise level, the accuracy of data acquisition in the 0–3 m detection range of the equipment was ensured. After the debugging was completed, the distance between the antenna and the lining surface was fixed to ensure the electromagnetic wave coupling effect in the detection area (at the central axis, 30 cm from both the upper and lower ends of the surrounding rock).

**Figure 2:**
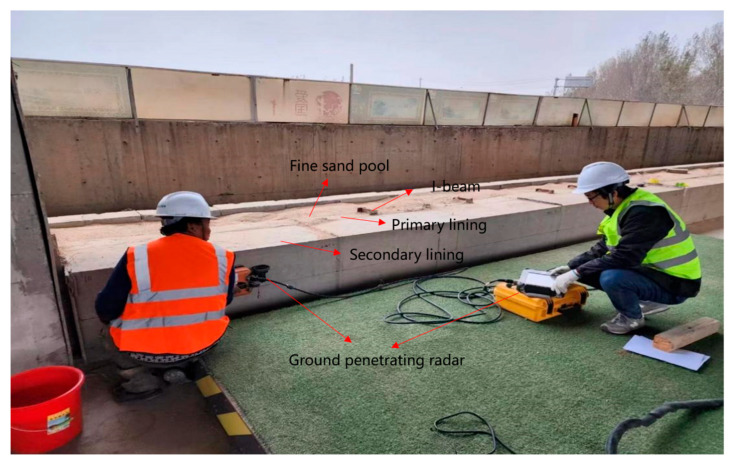
GPR Data Acquisition.

Baseline data acquisition was carried out in the defect-free area (Figure 2), and a 2 m long detection range was defined along the central axis of the lining. A 900 MHz high-frequency antenna was used to scan at a constant speed of 0.1 m/s, and each measurement line was repeated twice to obtain 20 groups of original waveform data. The data was saved in LTE format, and the GPS timestamp and environmental temperature, and humidity parameters were synchronously recorded to provide background references for subsequent defect analysis.

The data collection of defect working conditions was completed through four steps: positioning, embedding, filling, and measurement. Firstly, the simulated position of the defect was calibrated, and the embedding trench was excavated. The PVC cylinder was implanted until the bottom of the pipe was 10 cm lower than the radar detection area. Subsequently, water was injected into the cylinder to a height of 40 cm. After standing still, a 400 MHz high-frequency antenna of the geological radar was used to scan closely against the lining surface. The measurement was repeated twice for each group of working conditions. After the water injection data collection was completed, the liquid was pumped out, and crushed stones were filled in layers to the same height and vibrated and compacted. The scanning process was repeated. Finally, the burial depth of the PVC pipe was adjusted (0 cm, 17 cm, and 35 cm), and successively, the measurement steps of water injection and crushed stone filling were carried out. A total of 162 groups of valid data were obtained, which were stored according to the type of working conditions, and the low signal-to-noise ratio data were eliminated to ensure the reliability of the experimental results.

## 3. Analysis and Summary of Test Results

### 3.1. Radar Scan Image

In the lining model, PVC was used to set up voids, and water and sand were added to simulate different defects. Waterproof boards were set between the concrete lining and the sandpit, and steel mesh and I-beams were arranged at appropriate positions in the lining. The above content can be clearly distinguished from the image through the scanning of the ground-penetrating radar.

**Figure 3:**
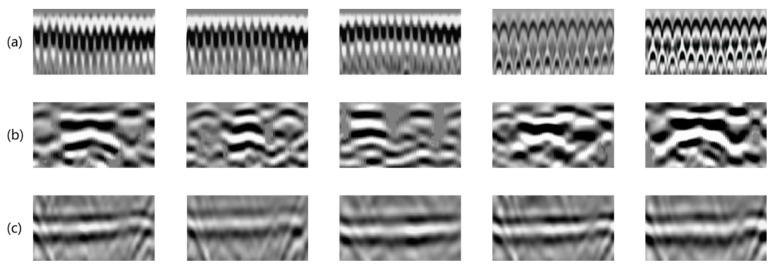
(**a**) Waveform of double-layer steel mesh; (**b**) I-beam waveform; (**c**) waterproof board between the primary support and the secondary lining.

As can be seen from Figure 3, the pre-arranged steel mesh, I-beams, and waterproof boards in the lining model can be well detected. As shown in Figure 3a, after the geological radar detects the steel mesh, its waveform will show dense short reflection signals, forming regular grid-like or parallel line-like arrangements. And due to its double-layer structure, two rows of reflected signals appear in the depth direction, with the spacing corresponding to the vertical distance between the two layers of steel mesh. Figure 3b shows the waveform form of the I-beam. Because of its special metal structure (the cross-section is “work” shape), the I-beam shows a significant hyperbolic feature in the radar profile, and the middle web may reflect a weak signal due to its small thickness. The dielectric constant of the waterproof board in Figure 3c differs significantly from that of the surrounding concrete, forming a continuous, horizontal, strongly reflective interface.

**Figure 4:**
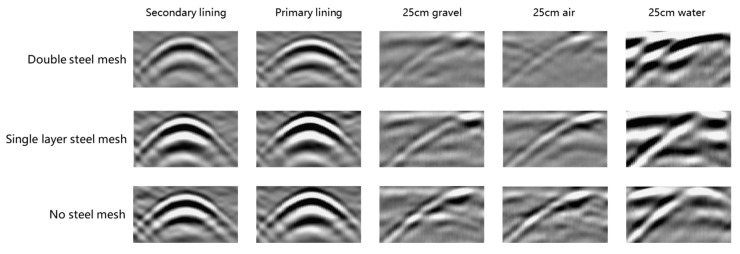
Geological radar defect simulation waveform diagram of different steel mesh configurations and media.

Figure 4 shows the corresponding radar waveforms of the ground radar under three types of steel mesh configurations, including secondary lining voids, initial support voids, and three types of medium simulated defects. By using a 25 cm diameter pvc pipe to simulate defect waveforms for analysis, the dielectric difference effect between the target body and the medium can be maximized. From the perspective of the influence of the steel mesh, the double-layer steel mesh, due to its strong electromagnetic shielding, significantly interferes with the radar waves. Single-layer steel mesh comes second. When there is no steel mesh, the reflection of defective targets is the clearest. From the perspective of medium differences, the dielectric constant of water is much higher than that of air and crushed stone, and the corresponding anti-harvesting signal amplitude is the largest, with the highest waveform recognition. The dielectric difference between crushed stone and air is relatively small, and the waveform amplitude is relatively mild. In conclusion, it is difficult to accurately determine the internal fillers of the surrounding rock void merely relying on waveform analysis. If it is necessary to clarify the category of the filling material, it is necessary to conduct an in-depth analysis of the radar waveform data with the help of time–frequency-domain analysis and other technologies.

### 3.2. Frequency-Domain Analysis

Time–frequency-domain analysis is a signal processing method that simultaneously utilizes time and frequency information, aiming to reveal the variation in the frequency characteristics of the signal over time. Traditional time-domain or frequency-domain methods can only provide information in a single dimension. However, time–frequency analysis, by combining the advantages of both, can effectively handle non-stationary signals, such as complex echoes in ground-penetrating radar signals. The frequency components of non-stationary signals change over time. Therefore, the echoes of ground-penetrating radar signals reflect the characteristics of different depths and materials at different times. Time-domain analysis is the basic method of geological radar signal processing. By directly observing the echo signal waveform of the geological radar, the arrival time, amplitude variation, and waveform shape of the signal can be obtained. The limitation of time-domain analysis lies in that it can only show the variation in signal amplitude over time but cannot clearly reveal the frequency components. Frequency-domain analysis mainly converts radar signals from the time domain to the frequency domain through Fourier transform, which can reveal the spectral characteristics of the signals. From this process, information such as spectral distribution, frequency band range, and attenuation characteristics can be obtained. The drawback of frequency-domain analysis lies in that it only provides the overall distribution of signal frequencies but loses the resolution of time, and is unable to describe the dynamic process of frequency variation over time. To make up for the deficiencies of time-domain and frequency-domain analysis, time–frequency-domain analysis combines the advantages of both and is capable of simultaneously describing the time-varying characteristics and frequency distribution of the signal.

In the identification of rock void defects by georadar, frequency-domain analysis, as the primary step, can give priority to revealing the response differences in different frequency components in the echo signal. Since the spectral structure of radar waves undergoes specific modulation and deformation when passing through filled media with different electromagnetic characteristics, the spectral characteristics can reflect key parameters such as the dielectric properties, absorption characteristics, and interface reflection capabilities of the fillers. Especially in complex environments with diverse types of fillers and weak electromagnetic differences, frequency-domain indicators can provide a more sensitive and stable discrimination basis. By giving priority to conducting spectral analysis, regions with significant differences in electromagnetic responses can be rapidly screened out as a whole, providing directional guidance and explanatory basis for subsequent time-domain waveform analysis, thereby achieving a more systematic and accurate identification of the types of substances in the void. The definition of the spectrum is as follows:

*P_k_* is the normalized power of the kth frequency component of the signal:

(1) $$P_{k}=\frac{{\left|X\left(f_{k}\right)\right|}^{2}}{\sum_{k=1}^{N}{\left|X\left(f_{k}\right)\right|}^{2}}$$

*X*(*f_k_*) is the Fourier transform value of the signal at frequency *f_k_*;

*N* is the total number of frequency points.

**Figure 5:**
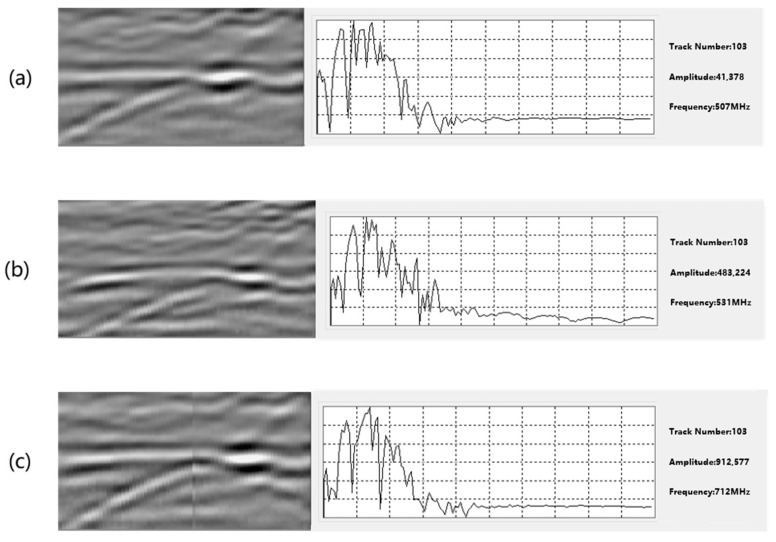
Different fillers for surrounding rock defects (the radar waveform and spectrum diagram of (**a**): crushed stone; (**b**): air; (**c**): water).

As shown in Figure 5, the peak of the spectrum when the gravel is filled is about 507 MHz, and the peak amplitude is only about 4.1 × 10^4^. The high-frequency component decays rapidly, and the spectral band is relatively narrow and flat, which indicates that the multipath scattering and microscopic void between the gravel particles have a significant dissipation effect on the high-frequency signal. In contrast, when air is filled, the peak frequency rises to approximately 531 MHz, the peak amplitude jumps to 4.8 × 10^5^, and the high-frequency bandwidth is significantly expanded, reflecting a large dielectric constant jump and low loss at the air–rock interface. This not only enables efficient reflection but also retains more high-frequency components. When the hollow is filled with water, the center of gravity of the spectrum further rises to approximately 712 MHz, and the peak amplitude increases to 9.1 × 10^5^. The high-frequency spectral lines become steep, and secondary peaks and valleys appear, indicating that the water fissure not only generates strong interfacial reflections but also enhances the high-frequency components through cavity resonance or waveguide effects.

### 3.3. Time-Domain Analysis

Ground-penetrating radar signals are records of the electromagnetic waves reflected by underground targets. Analyzing the statistical characteristics of the signals (such as amplitude and spectral entropy) helps reveal the characteristics of underground media, the reflection intensity of the targets, and the noise distribution.

Amplitude is the instantaneous value or maximum deviation value of a ground-penetrating radar signal. The magnitude of its value directly reflects the reflection intensity of the target. High amplitude usually corresponds to relatively strong reflection targets (such as steel mesh and I-beams in linings), while low amplitude corresponds to relatively weak reflection targets or noise. The formula of its root mean square amplitude is as follows:

(2) $$A_{RMS}=\sqrt{\frac{1}{N}\sum_{i=1}^{N}x_{i}^{2}}$$

**Figure 6:**
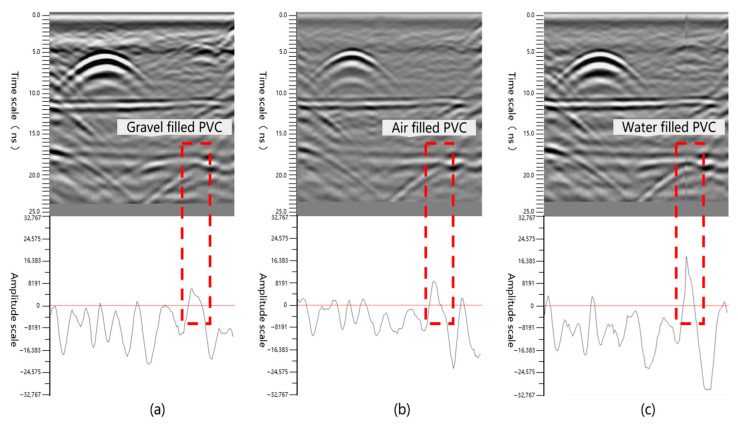
Different fillers for surrounding rock defects (radar waveform and amplitude diagram of (**a**): crushed stone; (**b**): air; (**c**): water).

Figure 6 shows the waveform diagram detected by the ground-penetrating radar and the amplitude distribution at different positions at the same depth. Judging from the characteristics of the amplitude curve in the figure, when PVC is filled with crushed stones, the amplitude fluctuation is relatively gentle (root mean square value 7241), the peak is small and dispersed. Due to the multiple scattering of crushed stone particles and microscopic voids, the reflected energy of radar waves is dispersed. When PVC is filled with air, the amplitude shows a high and sharp peak (root mean square value 9368), indicating that the dielectric constant difference between the air and the surrounding medium is large and the interface reflection is strong. When PVC is filled with water, the amplitude peak is higher (root mean square value 19,032), and the tailing is obvious. Due to the high dielectric constant of water, strong reflection occurs. Meanwhile, its absorption characteristics and multiple reflections make the signal attenuation complex. The numerical comparison shows that the root mean square value of the amplitude of water-filled defects is approximately twice that of air and 2.6 times that of crushed stones, further confirming the significant influence of dielectric difference on the reflection intensity. These differences in amplitude characteristics provide an intuitive basis for discriminating the filling substances in the defect based on amplitude, which is helpful for accurately identifying the types of surrounding rock defects.

In this study, in order to further determine the filling material inside the dehollowing in the time domain, from the LTE format B scan data generated by the georadar, the column number of the defect waveforms was precisely located based on the scanning distance or position index, and the corresponding time series data of the column were extracted to obtain a single A scan waveform. B scan is essentially a two-dimensional image composed of multiple A scan waveforms side by side in the spatial direction, while A scan reflects the electromagnetic wave amplitude characteristics of the radar antenna at a single spatial point over time (or depth). Since the reflection interface in the defect area usually presents special waveforms in the time-depth domain, direct analysis of the A scan can reveal microscopic features such as the arrival time difference, amplitude attenuation, and spectral distribution of the reflected signal, and thus more accurately characterize the location, geometry, and medium properties of the subsurface defect. Therefore, the in-depth analysis of the A scan at the defects in this thesis can not only supplement the qualitative description of the B scan visual observation, but also provide a reliable data basis for quantitative inversion and feature extraction.

**Figure 7:**
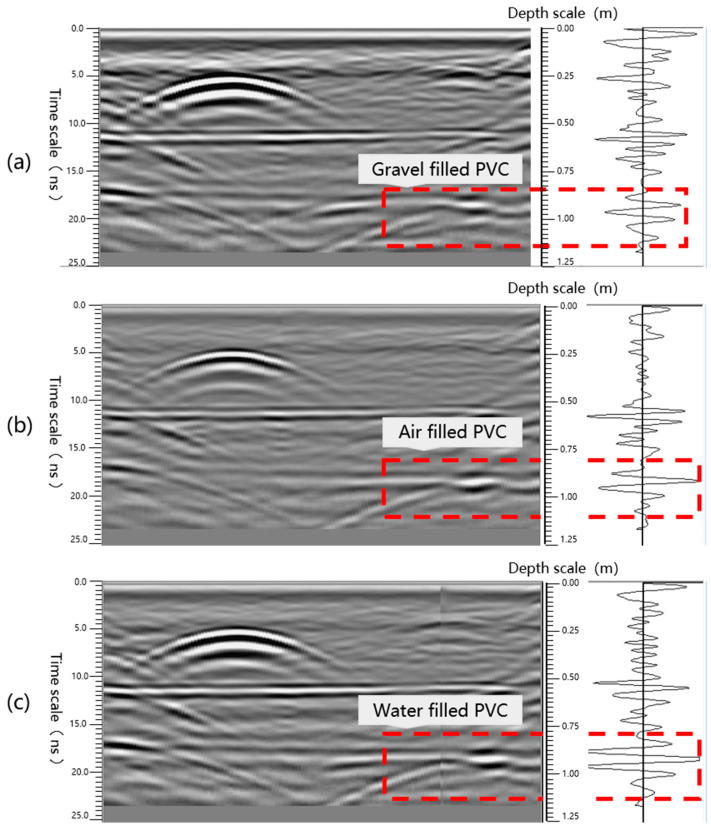
Different fillers for surrounding rock defects ((**a**): crushed stone; (**b**): Air; (**c**): water)) of radar waveform and A scan.

The three A scan curves in the Figure 7 exhibit the differential characteristics of three typical filling states in the time domain: When the tube is filled with water, the wave amplitude is the largest, the waveform is the steepest, and the trailing behind the wave and multiple echoes are the most obvious, reflecting the strong dielectric constant contrast at the water-PVC interface. Moreover, the higher absorption loss slows down the signal propagation speed and concentrates the energy in the void. When filled with air, the wave amplitude is centered, the waveform is sharp, and the attenuation is rapid, indicating that the contrast at the air–PVC interface is sufficient to produce obvious reflections. However, since air has almost no absorption, the echo energy is released rapidly, and no persistent tailing is formed. In contrast, the A scan filled with crushed stones presents the smallest wave amplitude and the widest waveform width. The subsequent echo energy is weak and dispersed, which is closely related to the signal dispersion and attenuation caused by multipath scattering and microscopic voids within the crushed stones.

In conclusion, through the joint analysis of the frequency domain and the time domain, the differences in electromagnetic responses among different filling media have been fully demonstrated. The frequency-domain analysis reveals the reflection and attenuation characteristics of different fillers on each frequency component of the radar wave, while the time-domain waveform reveals the delay characteristics and energy distribution pattern of the signal during the propagation process. The two complement and verify each other, making the discrimination of the types of fillers in the surrounding rock voids more physically based and reliable. Based on this joint analysis method, the identification of the types of filling media inside the voids can be effectively achieved, providing theoretical support for the subsequent assessment of defect properties and treatment strategies.

## 4. Conclusions

In this study, through the model test of ground-penetrating radar, the radar waveforms and frequency-domain characteristics of different filling media behind the tunnel lining were systematically analyzed, and the following main conclusions were formed:

(1) The characteristics of the defect waveforms are significantly differentiated: The radar signals of different filling media (crushed stones, air, and water) show differences in amplitude intensity and A scan signals in the time domain, while in the frequency domain, they are manifested as regular changes in the peak frequency, bandwidth, and energy distribution of the spectrum. Among them, the signal amplitude in the water-filled area is the highest, and the high-frequency component is prominent; the high-frequency component in the air-filled area is in the middle, and the energy in the crushed stone-filled area is dispersed, and the amplitude is gentle, providing an intuitive basis for the qualitative analysis of defects.

(2) The advantages of the joint analysis in the time–frequency domain are prominent: The time-domain A scan signal and amplitude can precisely describe the local reflection characteristics of defects (such as time difference in arrival and energy attenuation), while the frequency-domain analysis can explore the dielectric property differences in the medium through the spectral distribution. The combination of the two effectively solves the problem of insufficient discrimination ability for complex defects and improves the identification accuracy of filling material categories.

(3) Clear engineering application value: The typical defect waveform database and feature extraction method established through experiments have laid a data foundation for the subsequent application of deep learning algorithms in the automatic identification of tunnel defects. The research results can be directly applied to actual engineering detection. By analyzing the spectral peaks, amplitude attenuation, and time-domain morphology of radar signals, the filling types of voids and cracks behind the lining can be quickly determined, providing a scientific basis for the maintenance of tunnel structures.

The summarized waveform and spectral indicators provide a quantitative reference for on-site GPR interpretation. By analyzing parameters such as spectral peak frequency and amplitude attenuation, engineers can rapidly assess the type and severity of lining defects, thereby supporting timely maintenance decisions and improving the reliability of non-destructive tunnel evaluation. However, it should be noted that this study was conducted under controlled laboratory conditions using a full-scale physical model. Although this approach ensures high measurement accuracy and repeatability, it does not fully represent the complexity of real tunnel environments—such as uneven moisture distribution, surface irregularities, and ambient noise. Future research will, therefore, focus on extending the proposed method to field applications and validating its robustness under diverse in situ conditions.

In future work, the waveform-based discrimination framework established in this study can be further integrated with machine learning algorithms and field GPR platforms to realize automated feature extraction and intelligent identification of tunnel lining defects. This will provide technical support for rapid, non-destructive, and intelligent tunnel inspection.

## Figures and Tables

**Table 1:** Test operating conditions table.

Working Condition	Height (m)	Length (m)	Primary Lining/Secondary Lining (m)	I-Beam Model	Cavity Location	Steel Mesh
1	1.8	2.5	0.23/0.5	18	Primary lining	√
2	0.6	3.0	0.19/0.3	14	secondary lining	√
3	0.6	2.5	0.23/0.5	\	secondary lining	√
4	0.6	2.5	0.23/0.5	18	secondary lining	√
5	0.6	3.0	0.19/0.3	14	Primary lining	√
6	0.6	2.5	0.23/0.5	\	Primary lining	√
7	0.6	3.0	0.19/0.3	14	\	√
8	0.6	2.5	0.23/0.5	\	\	√
9	0.6	2.5	0.23/0.5	18	\	√
10	0.6	1.5	0.2/0.6	\	\	\

## Data Availability

The raw data supporting the conclusions of this article will be made available by the authors on request.
